# Supplementary material for: Occupational exposures and exacerbations of asthma and COPD—A general population study
Source: PLoS One. 2020 Dec 28;15(12):e0243826. doi: 10.1371/journal.pone.0243826 (PMC7769267; doi:10.1371/journal.pone.0243826)
Supplement: S1 Table — (DOCX) [file pone.0243826.s001.docx]

**Table S1. Overview of the methodology**

| **Step** | **Course of action** |
| --- | --- |
| 1 | Cohort studies conducted |
| 2 | Study population selected based on inclusion and exclusion criteria |
| 3 | Job codes held during follow-up obtained from the Danish Occupational Cohort database (DOC*X) |
| 4 | Imputation of missing job codes |
| 5 | Conversion of job exposure matrices (the Airborne Chemical Job Exposure Matrix (ACEJEM) and the Occupational Asthma-specific Job Exposure Matrix (OAsJEM)) to Statistics Denmark's Classification of Occupations (DISCO-88) |
| 6 | Exposure classes established |
| 7 | Job codes linked with the job exposure matrices for selected categories of exposure |
| 8 | Outcome variables collected from The Danish National Prescription Registry and The Danish National Patient Register |
| 9 | Analyses conducted |
