# Supplementary material for: Occupational exposures and exacerbations of asthma and COPD—A general population study
Source: PLoS One. 2020 Dec 28;15(12):e0243826. doi: 10.1371/journal.pone.0243826 (PMC7769267; doi:10.1371/journal.pone.0243826)
Supplement: S2 Table — (DOCX) [file pone.0243826.s002.docx]

**Table S2. Exposure classes combining level and proportion of exposure assigned by The Airborne Chemical Job Exposure Matrix**

|  |  | **Proportion** | | | |
| --- | --- | --- | --- | --- | --- |
|  |  | <5% | 5-19% | 20-49% | ≥50% |
| **Level** | Not exposed | Not exposed | Not exposed | Not exposed | Not exposed |
|  | Low | Not exposed | Low | Low | Low |
|  | Medium | Not exposed | Low | Low | High |
|  | High | Not exposed | Low | Low | High |
