# Supplementary material for: Occupational exposures and exacerbations of asthma and COPD—A general population study
Source: PLoS One. 2020 Dec 28;15(12):e0243826. doi: 10.1371/journal.pone.0243826 (PMC7769267; doi:10.1371/journal.pone.0243826)
Supplement: S3 Table — (DOCX) [file pone.0243826.s003.docx]

**Table S3. Exposure at study inclusion in study population and matched group**

|  | | | | | | |
| --- | --- | --- | --- | --- | --- | --- |
|  | **Study population** | | | **Matched* group of participants** | | |
|  | **Exposure, number (row-%)** | | | **Exposure, number (row-%)** | | |
| **ACE JEM** | Unexposed | Low | High | Unexposed | Low | High |
| Vapors, gases, dusts or fumes | 4,906 (63) | 2,184 (28) | 678 (9) | 15,143 (65) | 6,271 (27) | 1,851 (8) |
| Mineral dusts | 6,167 (79) | 1,189 (15) | 412 (5) | 18,731 (81) | 3,420 (15) | 1,114 (5) |
| Biological dusts | 6,368 (82) | 1,276 (16) | 124 (2) | 19,384 (83) | 3,517 (15) | 364 (2) |
| Gases&fumes | 7,236 (93) | 352 (5) | 180 (2) | 21,823 (94) | 915 (4) | 527 (2) |
|  |  |  |  |  |  |  |
| **OAsJEM** | Unexposed | Exposed |  | Unexposed | Exposed |  |
| High molecular weight sensitizer | 6,739 (87) | 1,029 (13) | - | 20,400 (88) | 2,865 (12) | - |
| Low molecular weight sensitizer | 6,633 (85) | 1,135 (15) | - | 19,981 (86) | 3,284 (14) | - |
| Irritants | 5,889 (76) | 1,879 (24) | - | 18,067 (78) | 5,198 (22) | - |
|  |  |  |  |  |  |  |
| *One-to-three matched controls based on sex, age at inclusion, smoking status, BMI, education and participation after the year 2000. Abbreviations: ACE JEM: The Airborne Chemical Job Exposure Matrix; OAsJEM: The Occupational Asthma-specific JEM | | | | | | |
