# Supplementary material for: Occupational exposures and exacerbations of asthma and COPD—A general population study
Source: PLoS One. 2020 Dec 28;15(12):e0243826. doi: 10.1371/journal.pone.0243826 (PMC7769267; doi:10.1371/journal.pone.0243826)
Supplement: S4 Table — (DOCX) [file pone.0243826.s004.docx]

**Table S4. Full Cox regression model with time varying exposure and age as underlying time scale**

|  | **Hazard ratio (95% CI)** |
| --- | --- |
| Vapours, gases, dusts or fumes |  |
| No | 1 (ref) |
| Low | 1.0 (0.8;1.1) |
| High | 1.0 (0.8;1.3) |
| Sex |  |
| Male | 1 (ref) |
| Female | **1.5 (1.3;1.8)** |
| Education* |  |
| Elementary | 1 (ref) |
| High School | 0.9 (0.8;1.2) |
| Academic | 0.8 (0.6;1.1) |
| Smoking* |  |
| Never | 1 (ref) |
| Former | 1.1 (0.9;1.3) |
| Current | 1.1 (0.9;1.4) |
| Body mass indexI* |  |
| <18.5 | 1.2 (0.6;2.3) |
| 18.5-24.9 | 1 (ref) |
| 25-29.9 | **1.3 (1.1;1.5)** |
| ≥30 | **1.5 (1.3;1.9)** |
| FEV_1_ % predicted* |  |
| ≥80% | 1 (ref) |
| <80% | **1.5 (1.3;1.8)** |
| Exacerbations one year prior to inclusion |  |
| No | 1 (ref) |
| ≥ 1 | **6.9 (5.6;8.5)** |
| * Status at baseline. Abbreviations; CI: confidence interval | |
