# Supplementary material for: Occupational exposures and exacerbations of asthma and COPD—A general population study
Source: PLoS One. 2020 Dec 28;15(12):e0243826. doi: 10.1371/journal.pone.0243826 (PMC7769267; doi:10.1371/journal.pone.0243826)
Supplement: S5 Table — (DOCX) [file pone.0243826.s005.docx]

**Table S5. Associations between exposure and exacerbations of self-reported asthma**

|  | **Events** | **Crude** | **Adjusted*** |
| --- | --- | --- | --- |
|  | ***Number*** | ***HR (95% CI)*** | ***HR (95% CI)*** |
| Vapors, gases, dusts or fumes |  |  |  |
| No | 355 | 1 (ref) | 1 (ref) |
| Low | 136 | 0.9 (0.7;1.1) | 0.9 (0.7;1.1) |
| High | 50 | **1.3 (1.0;1.7)** | 1.0 (0.8;1.4) |
| Mineral dusts |  |  |  |
| No | 437 | 1 (ref) | 1 (ref) |
| Low | 74 | 0.9 (0.7;1.2) | 1.0 (0.8;1.3) |
| High | 30 | 1.3 (0.9;1.8) | 1.0 (0.7;1.5) |
| Biological dusts |  |  |  |
| No | 449 | 1 (ref) | 1 (ref) |
| Low | 83 | 0.9 (0.8;1.2) | 0.8 (0.7;1.1) |
| High | 9 | NA | NA |
| Gases&fumes |  |  |  |
| No | 499 | 1 (ref) | 1 (ref) |
| Low | 28 | 1.4 (0.9;2.0) | **1.6 (1.1;2.3)** |
| High | 14 | 1.3 (0.8;2.2) | 1.0 (0.6;1.6) |
| High molecular weight sensitizer | |  |  |
| Unexposed | 476 | 1 (ref) | 1 (ref) |
| Exposed | 65 | 0.9 (0.7;1.2) | 0.8 (0.6;1.0) |
| Low molecular weight sensitizer |  |  |  |
| Unexposed | 466 | 1 (ref) | 1 (ref) |
| Exposed | 75 | 1.0 (0.7;1.2) | 0.9 (0.7;1.1) |
| Irritants |  |  |  |
| Unexposed | 409 | 1 (ref) | 1 (ref) |
| Exposed | 132 | 1.1 (0.9;1.4) | 1.0 (0.8;1.2) |
|  |  |  |  |
| Cox regression with time varying exposure and age as underlying time scale *adjusted for sex, education, smoking status, body mass index and FEV_1_ % predicted. Abbreviations; HR: hazard ratio; CI: confidence interval. | | | |
