# Supplementary material for: Occupational exposures and exacerbations of asthma and COPD—A general population study
Source: PLoS One. 2020 Dec 28;15(12):e0243826. doi: 10.1371/journal.pone.0243826 (PMC7769267; doi:10.1371/journal.pone.0243826)
Supplement: S6 Table — (DOCX) [file pone.0243826.s006.docx]

**Table S6. Associations between selected inhalant hazards and exacerbations in individuals with FEV_1_/FVC<0.70**

|  | **Events** | **Crude** | **Adjusted*** |
| --- | --- | --- | --- |
|  | ***Number*** | ***HR (95% CI)*** | ***HR (95% CI)*** |
| Vapors, gases, dusts or fumes |  |  |  |
| No | 278 | 1 (ref) | 1 (ref) |
| Low | 123 | 1.0 (0.8;1.2) | 1.0 (0.8;1.2) |
| High | 51 | 1.3 (1.0;1.7) | 1.1 (0.8;1.6) |
| Mineral dusts |  |  |  |
| No | 365 | 1 (ref) | 1 (ref) |
| Low | 56 | 0.9 (0.7;1.2) | 0.9 (0.7;1.3) |
| High | 31 | 1.2 (0.8;1.7) | 1.1 (0.8;1.7) |
| Biological dusts |  |  |  |
| No | 368 | 1 (ref) | 1 (ref) |
| Low | 78 | 1.1 (0.8;1.4) | 1.0 (0.8;1.3) |
| High | 6 | NA | NA |
| Gases&fumes |  |  |  |
| No | 418 | 1 (ref) | 1 (ref) |
| Low | 19 | 0.9 (0.6;1.4) | 1.0 (0.6;1.6) |
| High | 15 | 1.2 (0.7;2.0) | 1.1 (0.7;1.9) |
| High molecular weight sensitizer | |  |  |
| Unexposed | 390 | 1 (ref) | 1 (ref) |
| Exposed | 62 | 1.0 (0.8;1.3) | 0.9 (0.7;1.2) |
| Low molecular weight sensitizer | |  |  |
| Unexposed | 371 | 1 (ref) | 1 (ref) |
| Exposed | 81 | **1.3 (1.0;1.7)** | 1.3 (0.9;1.6) |
| Irritants |  |  |  |
| Unexposed | 317 | 1 (ref) | 1 (ref) |
| Exposed | 135 | **1.3 (1.1;1.6)** | 1.2 (0.9;1.5) |
|  |  |  |  |
| Cox regression with time varying exposure and age as underlying time scale *adjusted for sex, education, smoking status, body mass index and FEV_1_ % predicted. Abbreviations; HR: hazard ratio; CI: confidence interval. | | | |
