# Supplementary material for: Occupational exposures and exacerbations of asthma and COPD—A general population study
Source: PLoS One. 2020 Dec 28;15(12):e0243826. doi: 10.1371/journal.pone.0243826 (PMC7769267; doi:10.1371/journal.pone.0243826)
Supplement: Attachment — Submitted filename: Response to Reviewers.docx [file pone.0243826.s007.docx]

**Response to Reviewers**

Dear reviewer,

Thank you for investing time and effort into improving our manuscript and for your quick response. We were very pleased to receive your useful comments and suggestions, which are now all implemented in the paper.

**1. Describe, in general, the questionnaire used.**

Response: Thank you for addressing this. The questionnaire is now described in greater detail.

“At each round of examination, participants filled out a questionnaire, and completed a physical examination at a test center located at a public hospital in Copenhagen. The questionnaire was self-administered , concerning health status, lifestyle and socio-economic status, and was assessed by one of the investigators on the day of attendance.”

**2. In general, Methods section needs some additional information to be more comprehensible. Lines 61-62 – at which point was this measured? „Pre-bronchodilator FEV1 and FVC were measured at study inclusion and 62 repeated three times with the participant in a standing position.“ It’s unclear whether the authors have done it (i.e. approached these respondents and performed spirometry) or was it a question in questionnaire). If the respondents were approached, describe how did you approach the respondents and where did the examinations take place.**

Response: We agree that this part of the paper needed to be revised. Hopefully, it is now more comprehensible.

“The physical examination included spirometry. Pre-bronchodilator forced expiratory volume in 1 second (FEV_1_) and forced vital capacity (FVC) were measured by investigators and repeated three times with the participant in a standing position. The test was redone if the two closest trials differed by more than 5%, or the visual appearance of the spirometry tracing was unsatisfactory. A Vitalograph spirometer (Maids Moreton, Buckinghamshire) was used in The Copenhagen City Heart Study and by the first 14,624 individuals in the Copenhagen General Population Study, while an EasyOne Diagnostic Spirometer (ndd Medizintechnik, Switzerland) measured lung function in the remaining individuals.”

**3. A flowchart of the procedure would be out most helpful to the readers. What came first? Census – cohort study –Which occupations – the exposed group – ACEJEM / OAsJEM – statistical analysis… etc.**
Response: An overview of the process has been added to the supplementary tables to better illustrate the design.

**Table S1. Overview of the methodology**

| **Step** | **Course of action** |
| --- | --- |
| 1 | Cohort studies conducted |
| 2 | Study population selected based on inclusion and exclusion criteria |
| 3 | Job codes held during follow-up obtained from the Danish Occupational Cohort database (DOC*X) |
| 4 | Imputation of missing job codes |
| 5 | Conversion of job exposure matrices (the Airborne Chemical Job Exposure Matrix (ACEJEM) and the Occupational Asthma-specific Job Exposure Matrix (OAsJEM)) to Statistics Denmark's Classification of Occupations (DISCO-88) |
| 6 | Exposure classes established |
| 7 | Job codes linked with the job exposure matrices for selected categories of exposure |
| 8 | Outcome variables collected from The Danish National Prescription Registry and The Danish National Patient Register |
| 9 | Analyses conducted |

**4. Discussion –your angle on “the large decrease in most occupational inhalant exposures since the 1970s (40) combined with more safety equipment”… is sound and interesting. Please provide a short further explanation about PPE and other preventive measures in this subgroup (exposed to hazards). Which technical procedures and PPE are mandatory in Denmark regarding the selected hazards in your study? Were the data on PPE included in the questionnaire?**
Response: A very important issue. We have now elaborated on this.

“Improved technology and governmental regulation are important contributors to a large decrease in most occupational inhalant exposures since the 1970s (40) making findings plausible.”

…

“We were not able to account for the use of respiratory protective equipment (RPE), as this was not included in the ACE JEM or in the questionnaire. Legislation in Europe introduced in the 1980s has focused on adjustment of use of RPE as well as assessing its effectiveness, and thus RPE is now considered a last resort of protection. ”

**5. Reference 50 – seems to be incomplete**

Response: Thank you. We have corrected this.

We hope that the changes meet your expectations,

On behalf of co-authors,

Stinna Skaaby
